# Identifying and Overcoming Challenges in Developing Effective Treatments for Usher 1B: A Workshop Report

**DOI:** 10.1167/tvst.12.2.2

**Published:** 2023-02-01

**Authors:** Shannon E. Boye, Todd Durham, Amy Laster, Claire M. Gelfman, José-Alain Sahel

**Affiliations:** 1 Division of Cellular and Molecular Therapy, Department of Pediatrics, University of Florida, Gainesville, FL, USA; 2 Atsena Therapeutics, Inc., Durham, NC, USA; 3 Foundation Fighting Blindness, Columbia, MD, USA; 4 Sorbonne Université, INSERM, CNRS, Institut de la Vision, Paris, France; 5 Department of Ophthalmology, The University of Pittsburgh School of Medicine, Pittsburgh, PA, USA

**Keywords:** usher 1B workshop report, anatomy and pathology, gene therapy

## Abstract

**Purpose:**

To identify challenges and opportunities for the development of treatments for Usher syndrome (USH) type 1B.

**Methods:**

In September 2021, the Foundation Fighting Blindness hosted a virtual workshop of clinicians, academic and industry researchers, advocates, and affected individuals and their families to discuss the challenges and opportunities for USH1B treatment development.

**Results:**

The workshop began with insights from individuals affected by USH1B. Presentation topics included myosin VIIA protein function in the ear and eye and its role in disease pathology; challenges with the USH1B mouse model most used in disease research to date; new investigations into alternative disease models that may provide closer analogues to USH1B in the human retina, including retinal organoids and large animal models; and learnings from and limitations of available disease natural history data. Participants discussed the need for an open dialogue between researchers and regulators to design USH1B clinical trials with appropriate outcome measures of vision improvement, along with multimodal imaging of the retina and other testing approaches that can help inform trial designs. The workshop concluded with presentations and a roundtable reviewing emerging treatments, including USH1B-targeted genetic augmentation therapy and gene-agnostic approaches.

**Conclusions:**

Initiatives like this workshop are important to foster all stakeholders in support of achieving the shared goal of treating and curing USH1B.

**Translational Relevance:**

Presentations and discussions focused on overcoming disease modeling and clinical trial design challenges to facilitate development, testing, and implementation of effective USH1B treatments.

## Introduction

A genetic disorder that follows an autosomal recessive inheritance, Usher syndrome (USH) is a devastating diagnosis for affected individuals and their families. It is the most frequent cause of deaf–blindness, estimated to account for >50% of cases, as well as an estimated 5% of all congenital deafness cases and 18% of all retinitis pigmentosa (RP) cases.^1^^–^^5^ USH1B is a severe form of USH causing profound hearing loss at birth, progressive vision loss, and vestibular dysfunction.^1^^,^^5^ There is no treatment for USH1B currently, although several experimental therapies are in development, including treatments for RP and other USH subtypes with potential applicability to USH1B.

On September 13, 2021, the Foundation Fighting Blindness hosted a virtual workshop that brought together >120 stakeholders, including clinicians, researchers, industry representatives, advocates, and affected individuals and their families to discuss the current challenges and opportunities associated with developing USH1B treatments. Participants were invited to share perspectives and experiences, present research findings, and engage in cross-functional dialogue with the goal of defining the best path forward to successfully develop an effective treatment. Cochaired by José-Alain Sahel, MD, from the University of Pittsburgh Medical Center in Pennsylvania and the Institut de la Vision in Paris, France, and Shannon Boye, PhD, from the University of Florida in Gainesville and Atsena Therapeutics in Durham, North Carolina, the workshop consisted of a series of presentations interspersed with periods of discussion and concluded with a roundtable on emerging therapies. Key topics included the following:

• The importance of integrating patient perspectives into USH1B disease and treatment research
• Progress in understanding USH1B pathology
• Research into models that can further elucidate disease pathology and impact and be utilized to evaluate potential treatments
• The need for a more complete characterization of USH1B natural history
• The need to account for the multisensory dimensions of the disease
• Challenges in developing outcome measures for treatment trials
• Emerging treatments, including genetic augmentation therapies and gene-agnostic approaches

This article presents highlights from the workshop sessions and discussions.

## USH1B From the Perspective of Affected Individuals

Knowing the needs, concerns, and goals of individuals living with USH1B and understanding how this disease impacts their functioning and well-being are fundamental to successful treatment development. To provide workshop participants with insights from the community of individuals affected by USH1B, Todd Durham, PhD, of the Foundation Fighting Blindness in Columbia, Maryland, presented the results of a survey conducted by the Foundation Fighting Blindness among individuals recruited from the My Retina Tracker Registry and other networks (Table). The self-administered, online survey occurred from August 5 to September 6, 2021, and comprised 81 respondents reporting on the experiences of 97 affected individuals. Most respondents (*n* = 57) were adult caregivers of children with USH1B. Respondents’ most commonly reported visual symptoms were night blindness, sensitivity to light, and limited visual field, all of which were rated as moderate or severe by 65% to 80%. Almost all respondents reported deafness, poor balance, or other symptoms of vestibular dysfunction. Over half reported that further loss of vision was their biggest concern. Overall, respondents were moderately comfortable with participating in an USH1B treatment trial overseen by the Food and Drug Administration (FDA). Top questions about participating in such a trial involved treatment safety/expected side effects, the potential for worsening vision, and expected treatment benefits. Top motivations for trial participation were to stop disease progression, improve lost vision, and find a cure.

**Table.** Results of a Web-Based Survey of Individuals Affected by USH1B and Their Caregivers

Parameter	Respondents (*n*)	Result
Age of first clinical diagnosis, y	73	
Mean		8.1
Median (25th, 75th percentile)		6 (2, 12)
Age of first genetic diagnosis, y	73	
Mean		10.3
Median (25th, 75th percentile)		6 (2, 14)
Current BCVA in the worse eye, *n* (%)	66	
20/20 to 20/30		27 (41)
20/40 to 20/60		15 (23)
20/80 to 20/160		6 (9)
20/200 to 20/400		6 (9)
20/500 to 20/1000		2 (3)
Counting fingers, light perception, or no light perception		10 (15)
Self-rated severity of visual symptoms, *n* (%)		
Night blindness	70	
NA		6 (9)
Mild		8 (11)
Moderate		21 (30)
Severe		35 (50)
Sensitivity to light	68	
NA		7 (10)
Mild		16 (24)
Moderate		29 (43)
Severe		16 (24)
Limited visual field	68	
NA		12 (18)
Mild		12 (18)
Moderate		17 (25)
Severe		27 (40)
Poor visual acuity	68	
NA		17 (25)
Mild		22 (32)
Moderate		18 (26)
Severe		11 (16)
Lack of central vision	68	
NA		38 (56)
Mild		17 (25)
Moderate		7 (10)
Severe		6 (9)
Uncontrolled eye movement	65	
NA		53 (82)
Mild		5 (8)
Moderate		7 (11)
Severe		0
Other USH1B-related symptoms or physical health conditions, *n* (%)	55	
Deafness		28 (51)
Poor balance		22 (40)
Vestibular dysfunction unspecified		11 (20)
Autism		4 (7)
Cataracts		2 (4)
Cystoid macular edema		2 (4)
Vestibular migraine		2 (4)
Other headache		2 (4)
Low muscle strength/tone		2 (4)
Vestibular areflexia		2 (4)
Others (with one each)		10 (18)
Biggest source of worry related to USH1B, *n* (%)	78	
Total vision loss		24 (31)
Progressive vision loss		18 (23)
Unknown future		13 (17)
Participating and thriving		9 (12)
Mental health		8 (10)
Loss of independence/reliance on caregiver		7 (9)
Accidents/injury/safety		6 (8)
Inability to work		3 (4)
Not being able to communicate		2 (3)
Others (with one each)		5 (6)
Comfort level for participating in an FDA-overseen USH1B treatment clinical trial^a^	62	
Mean		58.4
Median (25th, 75th percentile)		60 (40, 80)
Top 1 to 3 questions about being in a trial, *n* (%)	?	
Safety/expected side effects		38
Will vision get worse?		18
Expected benefits		14
Able to participate in future trials?		7
How long is trial involvement?		7
Affect future access to treatment?		4
Trial appropriate for me/my child?		4
How is treatment administered?		4
Top 1 to 3 motivations (or desired outcomes) for trial participation, *n* (%)	58	
Stop progression		26 (45)
Improve lost vision		17 (29)
Finding a cure		13 (22)
Getting life back/independence		9 (16)
Advance treatments		8 (14)
Provide hope		5 (9)
Help others		5 (9)
Understand sign language better		1 (2)
See family		1 (2)

Self-administered, online survey in English conducted from August 5 to September 6, 2021. *N* = 81 respondents reporting on the experiences of 97 affected individuals (16 families had >1 affected individual). FDA, Food and Drug Administration; N/A, not applicable.

a 0 = not at all comfortable; 100 = totally comfortable.

One workshop participant, an adult affected by USH1B from the United States, spoke to workshop participants about his experience with the disease. He emphasized the unique challenges presented by the interplay between hearing loss and vision loss for people with USH1B. Because he is not completely deaf (due to cochlear implants) or completely blind, he said there is no large community of people with whom he can connect who share his experiences. With the increasing severity of vision loss, the impact of hearing loss also is becoming more severe—even though, objectively, his hearing has not changed—because he is no longer able to rely on his vision to compensate. “All of the things that are put forward as a solution to hearing problems,” he noted, “are based on you having good eyesight. All the problems with having blindness are combated with having good hearing.” Moreover, before it deteriorated, his vision was considered too good to fit the inclusion criteria for genetic treatment trials, highlighting a major limitation of current trial designs in reaching the goal of preventing vision loss. This affected adult's most important concern is knowing what the future holds for his ability to live independently and to start a family of his own.

Workshop participants also heard from two parents of children with USH1B, a father from the United States and a mother from Austria. The father spoke about the emotional toll USH1B has on parents and caregivers, noting that “the lack of control to be able to help your child is probably one of the most challenging things.” In searching for ways to contribute, he attributed the rarity of USH1B to the absence of an organization devoted solely to raising funds for treatment research. In response, he and his wife founded an organization that partners with the Foundation Fighting Blindness and is specifically focused on raising awareness and funds to support research into USH1B. Hailing from a small European country, the mother highlighted the importance of cross-country collaboration to establish research networks due to the rarity of USH1B and of ensuring European Union orphan drug regulations stimulate treatment development for such rare conditions. She also emphasized the urgent need for research into interventions that can be given at earlier stages when there is a “window of opportunity” to preserve vision. Interventions targeted for this stage capitalize on the other “window of opportunity” when adolescents with USH1B and their families are still motivated and engaged. Once vision loss accelerates, in her experience, patients and families begin to shut down emotionally, psychologically, and socially.

## Understanding USH1B Pathology

Identified in 1995, the gene associated with USH1B, *MYO7A*, was the first gene to be linked to USH.^6^^,^^7^ Since then, considerable progress has occurred in characterizing *MYO7A* mutations, the functions of the myosin VIIA protein encoded by the gene, and the impact of genetic mutations on protein expression and functioning.^1^^,^^2^^,^^7^^,^^8^^–^^12^ Aziz El-Amraoui, PhD, from Institut Pasteur, Institut de l'Audition in Paris, France, reviewed highlights of USH1B pathogenesis research and the investigation of various treatment approaches. Myosin VIIA is a member of a family of motor proteins with protein and molecular transport functions, as well as anchoring and tension functions, via interaction with actin.^7^^,^^12^^–^^16^ It is expressed in the sensory hair cells and mechanosensitive hair bundles of the inner ear and in the photoreceptor and retinal pigment epithelial (RPE) cells in the retina, as well as in other tissues.^10^^,^^16^^–^^19^ Myosin VIIA interacts with proteins associated with other USH subtypes, and together, these proteins form the Usher interactome.^7^^,^^20^ In the inner ear, myosin VIIA cooperates with these other proteins to ensure the cochlea and vestibular system hair bundles have the proper form and organization to function and is necessary for normal mechanoelectrical transduction in mature hair bundles. In USH1B, absence of functional myosin VIIA results in disorganized hair bundles and disrupted function, leading to profound hearing loss or deafness at birth and balance deficits throughout life.^14^^,^^15^^,^^21^^–^^25^ In the retina, it is thought that myosin VIIA deficiency leads to defective calyceal processes and impaired outer segment disc morphogenesis in photoreceptor cells, along with other abnormalities, such as melanosome and phagosome transport defects in RPE cells and delayed rhodopsin transport in photoreceptor cells. These defects are thought to underpin the progressive vision loss in USH1B.^8^^,^^13^^,^^26^^–^^30^ As the development and function of calyceal processes cannot be studied in rodent models, the latter are less relevant to understand the pathobiology of the disease and to validate potential therapies (see below).^26^^,^^27^

Additional information about USH1B pathogenesis relevant to treatment research came from David S. Williams, PhD, from the Stein Eye Institute and University of California in Los Angeles, California, who provided further details about transport defects in RPE and photoreceptor cells associated with *MYO7A* pathogenic variants. He noted that retinal disease from lack of myosin VIIA function may result from the cumulative effect of multiple defects, rather than from lack of a specific function. Claire Gelfman, PhD, of the Foundation Fighting Blindness, discussed what is known about USH genetics and how the large size of the *MYO7A* gene (6.6 kB) associated with USH1B complicates the development of gene therapies.^31^ Finally, Serge Picaud, PhD, Institut de la Vision, in Paris, France, presented research from his lab looking at a potential role for the Usher interactome in mechanotransduction in photoreceptor cone cells. This research showed the orientation of primate cone photoreceptor cilium occupies a decentered position at the edge of the cell, either on the side closest to the center of the retina, or on the opposite side and suggests that myosin VIIA may facilitate cone outer segment/inner segment alignment to the optic path.^32^

## Utility and Limits of USH1B Models

Much of our understanding of myosin VIIA's role in USH1B pathogenesis to date has been derived from experiments using mouse models, most commonly the *Myo7a^sh1/sh1^* (shaker1) model. As Dr. El-Amraoui discussed, mouse models for USH1B reproduce the characteristic early-onset hearing loss and vestibular dysfunction. The mouse model has allowed researchers to delineate how mutations in *MYO7A* impact cilia formation and function in the human inner ear because of the high conservation between the genetic pathways that regulate auditory perception in mice and humans. However, no USH1B mouse model shows retinal degeneration.^7^^,^^26^^,^^33^ Myosin VIIA localization and function in human and mouse RPE cells are comparable,^9^ but studies have found differences in photoreceptor structure and in relative expression of myosin VIIA.^7^^,^^26^^,^^33^ In humans, the calyceal processes form a ring around the photoreceptor outer segment, but this ring is lacking in mice.^26^ In addition, most myosin VIIA in the mouse retina is expressed in the RPE, whereas most mysoin VIIA in the monkey and human retina is expressed in the photoreceptors.^18^^,^^33^ Research into the extent and significance of these differences has sparked some debate. As Dr. Williams discussed, electron microscopy of mouse rod cells reveals the presence of one calyceal process.^34^ “If we are going to propose that the primate-like calyceal processes are essential for disk morphogenesis, then the narrative has to include why mouse and rat photoreceptors are able to control disc size, which they do, with only one calyceal process,” he said. Nevertheless, the lack of retinal degeneration in the USH1B mouse model presents a challenge in the essential preclinical development process, although the model is not entirely without utility. As Dr. El Amraoui said, “Even if, in terms of treatment validation outcomes, the eye is the ultimate goal, we can still use the information collected in the inner ear and use the inner ear as a window to look into the eye,” because of the short time frame needed for testing treatment effects and the well-characterized hearing and balance phenotypes in myosin VIIA–deficient mice. The mice, therefore, represent a model for evaluating potential cell and gene therapies for their ability to mitigate the hearing and vestibular defects, until myosin VIIA-deficient mice (or other relevant models) with progressive retinal degeneration are available.

The lack of retinal degeneration in the USH1B mouse model has led researchers to look elsewhere. At the workshop, Hannah Nonarath from the Medical College of Wisconsin in Milwaukee described the benefits and limitations of a zebrafish USH1B model. As a model system, zebrafish allow for easy genetic manipulation, are nearly transparent as embryos, and are small and inexpensive to maintain. In contrast to mice, zebrafish are diurnal, have calyceal processes, and present with a retinal phenotype. However, *MYO7A* is duplicated in zebrafish as *myo7aa* and *myo7ab*, which somewhat complicates the model. Ms. Nonarath's research has demonstrated that mutant zebrafish present with hair cell and vestibular defects and are sensitized to light damage, and adult *myo7ab^−^^/^^−^* zebrafish exhibit progressive loss of photoreceptors. Dr. Williams shared early results from a collaboration among researchers from his group and elsewhere seeking to characterize an USH1B pig model, as well as disease mechanism research from his lab using RPE derived from gene-edited human embryonic stem cells (hESCs) and induced pluripotent stem cells (iPSCs) from individuals affected by USH1B. He believes that melanosome dysregulation in the RPE is a result of disease and hopes to correlate this finding in patients using retinal imaging. David Gamm, MD, PhD, University of Wisconsin in Madison, also presented his work on modeling USH1B utilizing retinal organoids derived from iPSCs from individuals affected by USH1B. To date, his lab has shown myosin VIIA is expressed in these organoids but that this expression increases over time, which may make the model somewhat unwieldy. He has also validated antibodies for use in primate myosin VIIA expression studies and shown appropriate localization. Dr. Gamm also described USH1B nonsense mutation readthrough experiments being conducted at his lab using the iPSC-derived retinal organoid model in hopes of providing researchers with the ability to evaluate drugs for *MYO7A* readthrough at the preclinical level. Although initial readthrough experiments utilizing Western blot analysis found no detectable expression in this model or in an hESC photoreceptor reporter line with genetically engineered nonsense *MYO7A* mutations, research is continuing with the generation of new reporter lines and testing for readthrough via enhanced green fluorescent protein fluorescence.

At present, USH1B lacks a nonhuman primate (NHP) model, representing an important barrier to treatment development. Martha Neuringer, PhD, from the Oregon National Primate Research Center in Portland, presented an update on progress at her facility into developing such a model. She described the process of utilizing clustered regularly interspaced short palindromic repeats (CRISPR)–CRISPR-associated protein 9 (Cas9) technologies to edit *MYO7A* in monkey embryos and the birth of the first *MYO7A*-edited monkey, Mya, in May 2019. Unfortunately, Mya exhibited a mosaic pattern of editing (i.e., only some cells were edited) and lacked significant USH1B auditory and retinal phenotypes. Since then, a second infant, Gema, was born in November 2021. Gema showed a compound heterozygote pattern and demonstrated absence of auditory function at age 4 weeks. Development of the USH1B phenotypes in Gema will be followed longitudinally (Neuringer M, et al. *IOVS* 2022;63:ARVO E-abstract F0362). It is hoped that an NHP USH1B model will facilitate preclinical evaluation of treatments, as this model provides the closest analogue for the retinal structure and visual processing of humans.

Workshop attendees discussed the relative pros and cons of investing time and funding into further development of these and other alternatives to the shaker1 mouse USH1B model. Dr. El-Amraoui noted that although an amphibian model is possible with gene editing, large animal models represent a better use of resources because of the greater resemblance to the human condition. Because the pig eye has similar dimensions to that of humans, this model may hold more promise. In the NHP model, Dr. Neuringer stressed the need for increasing embryo and infant numbers as the biggest hurdle for achieving accurate USH1B phenotype expression and evaluating therapeutic approaches. Organoids grown from patient cells, what Dr. Neuringer termed “retina in a dish,” are easier to develop than animal models, can recapitulate a retinal degenerative phenotype, and may be useful in the earliest treatment testing stages. Currently, however, organoids do not represent fully developed retinas and do not permit the study of retinal cell changes in the context of the complete optical system. “The structure of the [photoreceptor] outer segment is key in this disease,” noted Dr. Sahel. “If there is no interaction of the outer segment with the RPE, outer segments may not develop. However, there are plans by several research groups to codevelop the RPE and organoids together.”

## Characterizing USH1B Natural History

Natural history studies for rare diseases like USH1B can be difficult to conduct but are highly relevant to treatment research. As Dr. Durham discussed in his analysis of USH1B natural history studies in the literature, these studies help define variables that correlate with disease onset, progression, and outcomes and identify the patient population most appropriate for an intervention. Natural history studies also provide data used to develop disease progression models, which inform clinical study simulations as part of model-based drug development. In his analysis, Dr. Durham found that the suitability of current USH1B natural history studies for informing clinical study design varies, and additional observational studies investigating the rate and nature of USH1B disease progression may be necessary, potentially incorporating measures of static perimetry, fundus-guided microperimetry, and dark-adapted visual fields. This would build upon currently available data that show there is an opportunity to retain or slow progression of peripheral vision loss for individuals affected by USH1B in the first four decades of life.^35^^–^^37^

Workshop participants also heard from Isabelle Audo, MD, PhD, from the Sorbonne and CHNO of Quinze-Vingts, Paris, France, who presented results from a study at her center investigating the structural and functional retinal changes with age in a cohort of 53 individuals with USH1B with rod-cone dystrophy to attempt to model disease progression. The researchers also sought to correlate *MYO7A* genotype in these individuals (50 distinct mutations with four novel changes) with the USH1B phenotype. Over a mean follow-up of 4 years (range, 0–15) for best-corrected visual acuity (BCVA) measures and 2.5 years (range, 0–16) for visual field measures, the study showed visual acuity and field regression worsened with age. In contrast, the pattern of structural changes associated with the disease course varied and was not associated with age of the individual. There also was no correlation between *MYO7A* mutations in specific functional domains and disease severity, although cohort size likely impacted the study's findings. These results suggest USH1B prognosis predictions should be made on a case-by-case basis, which may impact the design of treatment studies.

## Determining USH1B Clinical Trial Outcome Measures

“To have a successful clinical trial, we have to find a balance between the things we, as scientists, might want to test and the things that regulators really care about,” said Mark Pennesi, MD, PhD, from the Casey Eye Institute in Portland, Oregon. His presentation reviewed the structural and functional vision tests that can be used to measure the different dimensions that combine to form functional vision, which has the greatest impact on individuals’ lives, as well as the outcome measures and performance thresholds regulators currently require for clinical studies addressing inherited retinal degenerations (including USH1B). He noted that, in general, some of these measures and thresholds are based on glaucoma treatment studies and may not translate well to studies for USH1B, because they set the bar too high. In other words, visual improvement that is lower than what regulators currently require to approve an USH1B treatment may actually be clinically meaningful. Therefore, even though there are currently many different methods of evaluating vision, further work is needed to define additional outcome measures, including patient-reported outcomes, and end points that are sensitive, predictive, and accepted by regulators. Dr. Pennesi said the research community has an important role in informing regulators what the meaning of these tests are and what an improvement in these tests really means to patients. Workshop participants discussed potential ways to work with regulators on setting appropriate benchmarks for evaluating treatment in USH1B clinical trials, including incorporating insights from patients.

Research that can inform development of appropriate outcome measures is ongoing. Jacque Duncan, MD, University of California, San Francisco, presented the results of a small imaging study she and her colleagues conducted to attempt to characterize retinal, RPE, and choriocapillaris structure in individuals with USH1B. These studies utilized multimodal, high-resolution imaging modalities, including color fundus photos, spectral domain optical coherence tomography, short-wavelength autofluorescence, swept-source optical coherence tomography angiography, and adaptive optics scanning laser ophthalmoscopy. These techniques revealed that loss of the ellipsoid zone corresponds to areas of visual field impairment in individuals affected by USH1B. Autofluorescence highlighted the central location of functional RPE cells, and findings correlated with visual field margins. Adaptive optics imaging showed cones were well preserved in the central retina, and cone spacing was generally normal at the majority of regions studied in individuals with preserved maculas. However, cone loss became more pronounced in regions further from the fovea. Patients with USH1B in this study maintained normal choriocapillaris flow and good macular structure for treatment. The findings indicate that imaging cones at the margin of degeneration and RPE cells may provide a sensitive measure of disease progression and outcomes. Separately, Dr. Picaud discussed findings from studies conducted at his center concerning cone degeneration and phototoxicity in USH models showing that filtering out blue-violet light may be beneficial in preventing light toxicity.

## Emerging USH1B Treatment Approaches

### Genetic Augmentation Therapies

As previously mentioned, at 6.6 kb,^31^ the *MYO7A* coding sequence is relatively large. Its size precludes the use of standard adeno-associated viruses (AAVs) as vectors for USH1B-targeted genetic augmentation therapy due to their relatively small cargo capacity of about 5 kb.^38^^–^^40^ Workshop participants heard from representatives of two groups of researchers working to develop dual AAV vectors, a technology based on the inherent ability of two AAV genomes to undergo concatemerization and homologous recombination. With a dual AAV vector, the *MYO7A* coding sequence is divided into two pieces, and each piece is carried by one AAV. Via a region of complementary sequence shared between these two pieces, they are joined back together through homologous recombination to allow expression of full-length *MYO7A* in retinal cells and, ultimately, generation of a full-length protein.^41^^–^^43^ Alberto Auricchio, MD, from the Telethon Institute of Genetics and Medicine (TIGEM) in Naples, Italy, presented research that led to development of the hybrid dual AAV vector serotype 8 (AAV.8.*hMYO7A*), which has been shown to result in reconstitution of full-length myosin VIIA to therapeutic levels in the USH1B shaker1 mouse model.^41^ He reviewed work to date from the UshTher initiative in Europe, including toxicity and Good Manufacturing Practice lot manufacture and release testing, a dose–response study in shaker1 mice, and nonclinical studies in NHPs. He presented a timeline toward submitting the Investigational Medicine Product Dossier to regulators and moving to a clinical trial as early as 2022. Dr. Boye provided an overview of research from the USH1B genetic therapy development program at Atsena Therapeutics that is also investigating dual AAV vectors for use in this capacity. Her team has optimized hybrid vectors by altering split points between the front and back half vectors, as well as codon modifying the front half vector sequence to prevent production of truncated protein. They also have optimized overlap dual AAV vectors, a platform that relies on slightly different methods of recombination, to efficiently produce full-length *MYO7A*. She reported Atsena Therapeutics is currently conducting testing in mouse and NHP models to determine an optimal candidate vector and, from there, will move into studies to enable an investigational new drug application and a clinical trial. Hybrid dual AAV vector systems developed by the UshTher initiative and Atsena also have been shown to have potential to treat the vestibular dysfunction in USH1B when tested in the shaker1 mouse model. A possible caveat to this research: the dual AAV vector–based genetic augmentation therapy systems Dr. Auricchio's organization is researching contain *MYO7A* transcript variant 1, which, as Dr. Williams pointed out, is not as common as variant 2 in the human retina.^16^^,^^44^ Dr. Boye's organization is employing a version that encompasses properties of transcript variants 1 and 2. Both have shown the ability to correct melanosome migration in the shaker1 mouse. However, the significance of the different isoforms on retinal function in primates is unknown.

In a separate roundtable on emerging therapies, Atsena Therapeutics’ chief executive officer, Patrick Ritschel, presented additional information about the company, and representatives from two additional companies also provided updates about gene-based therapies whose approaches and mechanisms may be of relevance for the development of USH1B treatments. Vijay Modur, MBBS, PhD, from Eloxx Pharmaceuticals in Watertown, Massachusetts, discussed research being conducted at his company into the potential of eukaryotic ribosomal selective glycoside therapy. This technology may be used to address defects in protein translation associated with nonsense mutations and provide functional gene therapy via premature stop codon readthrough. Aniz Girach, MD, from ProQR Therapeutics in Leiden, the Netherlands, presented results from the STELLAR phase 1/2 clinical trial (NCT03780257) of QR-421a, a first-in-class RNA therapy that targets exon 13 mutations in individuals with USH2A with the aim of preventing blindness. This agent was shown to be safe and well tolerated, with no serious adverse events. In individuals with progressive visual acuity loss, stabilization of BCVA was observed in the treated eye, whereas vision declined in the untreated eye. Individuals with less advanced vision loss showed improvement on static perimetry (unpublished data).

### Gene-Agnostic Approaches

From Fort Worth, Texas–based Nacuity Pharmaceuticals, Inc., Halden Conner, MBA, and Jami Kern, MBA, PhD, provided an overview of the antioxidative molecule N-acetylcysteine-amide (NPI-001) being developed by their company. The oral, gene-agnostic drug is designed to slow vision loss by reducing oxidative stress. A 24-month clinical trial for NPI-001 enrolling 48 participants with USH (all forms) was initiated in Australia in January 2020 (NCT04355689), and a US trial for RP is planned in 2023.

## Summary

The progressive nature of retinal disease in USH1B underpins the urgency of affected individuals, particularly parents of young children and individuals in the earlier stages of vision loss, to accelerate treatment development. The presentations and discussions at this workshop provided a clearer picture of areas where researchers have made progress, challenges that remain, and reasons to hope that effective treatments may be on the horizon. Because it is a rare disease, cooperation and collaboration among stakeholders have an outsized impact on the success of these efforts. Initiatives like this workshop are important to foster communication between affected individuals, researchers, and industry representatives in support of achieving the shared goal of treating and curing USH1B.

## References

[1] Ferrari S, Di Iorio E, Barbaro V, Ponzin D, Sorrentino FS, Parmeggiani F. Retinitis pigmentosa: genes and disease mechanisms. *Curr Genomics*. 2011; 12(4): 238–249. 22131869 10.2174/138920211795860107 PMC3131731

[2] Millán JM, Aller E, Jaijo T, Blanco-Kelly F, Gimenez-Pardo A, Ayuso C. An update on the genetics of usher syndrome. *J Ophthalmol*. 2011; 2011: 417217. 21234346 10.1155/2011/417217 PMC3017948

[3] Boughman JA, Vernon M, Shaver KA. Usher syndrome: definition and estimate of prevalence from two high-risk populations. *J Chronic Dis*. 1983; 36(8): 595–603. 6885960 10.1016/0021-9681(83)90147-9

[4] Marazita ML, Ploughman LM, Rawlings B, Remington E, Arnos KS, Nance WE. Genetic epidemiological studies of early-onset deafness in the U.S. school-age population. *Am J Med Genet*. 1993; 46(5): 486–491. 8322805 10.1002/ajmg.1320460504

[5] Toms M, Pagarkar W, Moosajee M. Usher syndrome: clinical features, molecular genetics and advancing therapeutics. *Ther Adv Ophthalmol*. 2020; 12: 1–19. 10.1177/2515841420952194 PMC7502997 32995707

[6] Weil D, Blanchard S, Kaplan J, et al. Defective myosin VIIA gene responsible for Usher syndrome type 1B. *Nature*. 1995; 374(6517): 60–61. 7870171 10.1038/374060a0

[7] Fuster-García C, García-Bohórquez B, Rodríguez-Muñoz A, et al. Usher syndrome: genetics of a human ciliopathy. *Int J Mol Sci*. 2021; 22(13): 6723. 34201633 10.3390/ijms22136723 PMC8268283

[8] Gibbs D, Kitamoto J, Williams DS. Abnormal phagocytosis by retinal pigmented epithelium that lacks myosin VIIa, the Usher syndrome 1B protein. *Proc Natl Acad Sci USA*. 2003; 100(11): 6481–6486. 12743369 10.1073/pnas.1130432100 PMC164472

[9] Gibbs D, Diemer T, Khanobdee K, Hu J, Bok D, Williams DS. Function of MYO7A in the human RPE and the validity of shaker1 mice as a model for Usher syndrome 1B. *Invest Ophthalmol Vis Sci*. 2010; 51(2): 1130–1135. 19643958 10.1167/iovs.09-4032 PMC2868451

[10] Hasson T, Heintzelman MB, Santos-Sacchi J, Corey DP, Mooseker MS. Expression in cochlea and retina of myosin VIIa, the gene product defective in Usher syndrome type 1B. *Proc Natl Acad Sci USA*. 1995; 92(21): 9815–9819. 7568224 10.1073/pnas.92.21.9815 PMC40893

[11] Adato A, Weil D, Kalinski H, et al. Mutation profile of all 49 exons of the human myosin VIIA gene, and haplotype analysis, in Usher 1B families from diverse origins. *Am J Hum Genet*. 1997; 61(4): 813–821. 9382091 10.1086/514899 PMC1716000

[12] Inoue A, Ikebe M. Characterization of the motor activity of mammalian myosin VIIA. *J Biol Chem*. 2003; 278(7): 5478–5487. 12466270 10.1074/jbc.M210489200

[13] Wolfrum U, Schmitt A. Rhodopsin transport in the membrane of the connecting cilium of mammalian photoreceptor cells. *Cell Motil Cytoskeleton*. 2000; 46(2): 95–107. 10891855 10.1002/1097-0169(200006)46:2<95::AID-CM2>3.0.CO;2-Q

[14] Boëda B, El-Amraoui A, Bahloul A, et al. Myosin VIIa, harmonin and cadherin 23, three Usher I gene products that cooperate to shape the sensory hair cell bundle. *EMBO J*. 2002; 21(24): 6689–6699. 12485990 10.1093/emboj/cdf689 PMC139109

[15] El-Amraoui A, Petit C. Usher I syndrome: unravelling the mechanisms that underlie the cohesion of the growing hair bundle in inner ear sensory cells. *J Cell Sci*. 2005; 118(pt 20): 4593–4603. 16219682 10.1242/jcs.02636

[16] Li S, Mecca A, Kim J, et al. Myosin-VIIa is expressed in multiple isoforms and essential for tensioning the hair cell mechanotransduction complex. *Nat Commun*. 2020; 11(1): 2066. 32350269 10.1038/s41467-020-15936-z PMC7190839

[17] Liu X, Vansant G, Udovichenko IP, Wolfrum U, Williams DS. Myosin VIIa, the product of the Usher 1B syndrome gene, is concentrated in the connecting cilia of photoreceptor cells. *Cell Motil Cytoskeleton*. 1997; 37(3): 240–252. 9227854 10.1002/(SICI)1097-0169(1997)37:3<240::AID-CM6>3.0.CO;2-A

[18] El-Amraoui A, Sahly I, Picaud S, Sahel J, Abitbol M, Petit C. Human Usher 1B/mouse shaker-1: the retinal phenotype discrepancy explained by the presence/absence of myosin VIIA in the photoreceptor cells. *Hum Mol Genet*. 1996; 5(8): 1171–1178. 8842737 10.1093/hmg/5.8.1171

[19] Reiners J, Reidel B, El-Amraoui A, et al. Differential distribution of harmonin isoforms and their possible role in Usher-1 protein complexes in mammalian photoreceptor cells. *Invest Ophthalmol Vis Sci*. 2003; 44(11): 5006–5015. 14578428 10.1167/iovs.03-0483

[20] Weil D, El-Amraoui A, Masmoudi S, et al. Usher syndrome type I G (USH1G) is caused by mutations in the gene encoding SANS, a protein that associates with the USH1C protein, harmonin. *Hum Mol Genet*. 2003; 12(5): 463–471. 12588794 10.1093/hmg/ddg051

[21] Küssel-Andermann P, El-Amraoui A, Safieddine S, et al. Vezatin, a novel transmembrane protein, bridges myosin VIIA to the cadherin-catenins complex. *EMBO J*. 2000; 19(22): 6020–6029. 11080149 10.1093/emboj/19.22.6020 PMC305826

[22] Adato A, Michel V, Kikkawa Y, et al. Interactions in the network of Usher syndrome type 1 proteins. *Hum Mol Genet*. 2005; 14(3): 347–356. 15590703 10.1093/hmg/ddi031

[23] Michel V, Goodyear RJ, Weil D, et al. Cadherin 23 is a component of the transient lateral links in the developing hair bundles of cochlear sensory cells. *Dev Biol*. 2005; 280(2): 281–294. 15882573 10.1016/j.ydbio.2005.01.014

[24] Lefèvre G, Michel V, Weil D, et al. A core cochlear phenotype in USH1 mouse mutants implicates fibrous links of the hair bundle in its cohesion, orientation and differential growth. *Development*. 2008; 135(8): 1427–1437. 18339676 10.1242/dev.012922

[25] Bahloul A, Michel V, Hardelin J-P, et al. Cadherin-23, myosin VIIa and harmonin, encoded by Usher syndrome type I genes, form a ternary complex and interact with membrane phospholipids. *Hum Mol Genet*. 2010; 19(18): 3557–3565. 20639393 10.1093/hmg/ddq271 PMC2928128

[26] Sahly I, Dufour E, Schietroma C, et al. Localization of Usher 1 proteins to the photoreceptor calyceal processes, which are absent from mice. *J Cell Biol*. 2012; 199(2): 381–399. 23045546 10.1083/jcb.201202012 PMC3471240

[27] Schietroma C, Parain K, Estivalet A, et al. Usher syndrome type 1-associated cadherins shape the photoreceptor outer segment. *J Cell Biol*. 2017; 216(6): 1849–1864. 28495838 10.1083/jcb.201612030 PMC5461027

[28] Liu X, Ondek B, Williams DS. Mutant myosin VIIa causes defective melanosome distribution in the RPE of shaker-1 mice. *Nat Genet*. 1998; 19(2): 117–118. 9620764 10.1038/470

[29] Liu X, Udovichenko IP, Brown SDM, Steel KP, Williams DS. Myosin VIIa participates in opsin transport through the photoreceptor cilium. *J Neurosci*. 1999; 19(15): 6267–6274. 10414956 10.1523/JNEUROSCI.19-15-06267.1999 PMC6782817

[30] Gibbs D, Azarian SM, Lillo C, et al. Role of myosin VIIa and Rab27a in the motility and localization of RPE melanosomes. *J Cell Sci*. 2004; 117(pt 26): 6473–6483. 15572405 10.1242/jcs.01580 PMC2942070

[31] Xin F, Nguyen Huu VA, Duan Y, et al. Clinical applications of retinal gene therapies. *Precision Clin Med*. 2018; 1(1): 5–20. 10.1093/pcmedi/pby004 PMC8982485 35694125

[32] Verschueren A, Boucherit L, Ferrari U, et al. Planar polarity in primate cone photoreceptors: a potential role in Stiles Crawford effect phototropism. *Commun Biol*. 2022; 5(1): 89. 35075261 10.1038/s42003-021-02998-y PMC8786850

[33] Calabro KR, Boye SL, Choudhury S, et al. A novel mouse model of MYO7A USH1B reveals auditory and visual system haploinsufficiencies. *Front Neurosci*. 2019; 13: 1255. 31824252 10.3389/fnins.2019.01255 PMC6883748

[34] Volland S, Hughes LC, Kong C, et al. Three-dimensional organization of nascent rod outer segment disk membranes. *Proc Natl Acad Sci USA*. 2015; 112(48): 14870–14875. 26578801 10.1073/pnas.1516309112 PMC4672767

[35] Jacobson SG, Cideciyan AV, Gibbs D, et al. Retinal disease course in Usher syndrome 1B due to MYO7A mutations. *Invest Ophthalmol Vis Sci*. 2011; 52(11): 7924–7936. 21873662 10.1167/iovs.11-8313 PMC3263772

[36] Lenassi E, Saihan Z, Cipriani V, et al. Natural history and retinal structure in patients with Usher syndrome type 1 owing to MYO7A mutation. *Ophthalmology*. 2014; 121(2): 580–587. 24199935 10.1016/j.ophtha.2013.09.017

[37] Testa F, Melillo P, Bonnet C, et al. Clinical presentation and disease course of Usher syndrome because of mutations in MYO7A or USH2A. *Retina*. 2017; 37(8): 1581–1590. 27828912 10.1097/IAE.0000000000001389

[38] Grieger JC, Samulski RJ. Packaging capacity of adeno-associated virus serotypes: impact of larger genomes on infectivity and postentry steps. *J Virol*. 2005; 79(15): 9933–9944. 16014954 10.1128/JVI.79.15.9933-9944.2005 PMC1181570

[39] Lopes VS, Williams DS. Gene therapy for the retinal degeneration of Usher syndrome caused by mutations in MYO7A. *Cold Spring Harb Perspect Med*. 2015; 5(6): a017319. 25605753 10.1101/cshperspect.a017319 PMC4448706

[40] French LS, Mellough CB, Chen FK, Carvalho LS. A review of gene, drug and cell-based therapies for Usher syndrome. *Front Cell Neurosci*. 2020; 14: 183. 32733204 10.3389/fncel.2020.00183 PMC7363968

[41] Trapani I, Colella P, Sommella A, et al. Effective delivery of large genes to the retina by dual AAV vectors. *EMBO Mol Med*. 2014; 6(2): 194–211. 24150896 10.1002/emmm.201302948 PMC3927955

[42] Colella P, Trapani I, Cesi G, et al. Efficient gene delivery to the cone-enriched pig retina by dual AAV vectors. *Gene Ther*. 2014; 21(4): 450–456. 24572793 10.1038/gt.2014.8

[43] Trapani I, Toriello E, de Simone S, et al. Improved dual AAV vectors with reduced expression of truncated proteins are safe and effective in the retina of a mouse model of Stargardt disease. *Hum Mol Genet*. 2015; 24(23): 6811–6825. 26420842 10.1093/hmg/ddv386 PMC4634381

[44] Weil D, Levy G, Sahly I, et al. Human myosin VIIA responsible for the Usher 1B syndrome: a predicted membrane-associated motor protein expressed in developing sensory epithelia. *Proc Natl Acad Sci USA*. 1996; 93(8): 3232–3237. 8622919 10.1073/pnas.93.8.3232 PMC39588

